# Collectanea: Pathology and Practice

**Published:** 1835-01-01

**Authors:** 


### COLLECTANEA.

### PATHOLOGY AND PRACTICE.

### ANIMAL MAGNETISM.

WE have received an ingenious communication on this subject from Dr. STANLEY, of Brighton, which we do not publish, for the following reason. Dr. Stanley's paper mentions, with great brevity, that he has magnetized some persons, but it is chiefly occupied with the theory of the art. He supposes that slight galvanic shocks are thrown out by the fingers of the magnetizer; a theory which is certainly supported by some late discoveries, showing that the acid and alkaline secretions of different parts of the human body form a kind of voltaic pile. But it is not a theory of animal magnetism, however plausible, that is now wanted,—we require facts. Let Dr. Stanley come into our hospitals, and demand permission to cure a dozen patients by mesmerism: this will readily be granted; and, when he has convinced a number of hospital physicians and surgeons of the reality of the art, we will answer for the rest of the profession. That is to say, we will answer for every one except Dr. Bostock; for he tells us, "The conclusion that forces itself irresistibly on the mind is, that no medical testimony is insufficient to establish a fact which is in itself incredible, and that this previous incredibility can only be ascertained by an extensive and accurate knowledge of the functions and properties of the living body, both mental and corporeal, in all its modifications, and under all circumstances, and by a correct and careful generalization of the knowledge thus obtained." (*History of Medicine*, cap. xiii. *Cyclopædia of Pract. Med.* part xxii.)

Dr. Stanley is quite right when he says that we have no prejudices against animal magnetism; and we repeat that we wish, with Dr. Pritchard, that the art could be introduced into this country. But, that this may happen, it is necessary that everything should be done above board, and free from all suspicion, from all possibility of collusion. People begin to think that we have been tantalized long enough with talk about animal magnetism, and ask for facts. The mere account of foreign cases is not sufficient. The sober good sense of British practitioners requires that what was done at Paris or Vienna should be capable of repetition in London. If the phenomena cannot be transplanted to this colder climate, they are at any rate of no practical importance to us.

### CASE OF APPARENT HÆMOPTYSIS CAUSED BY A LEECH.

A shepherd had been spitting blood for eighteen days, and, being tired of taking the pectoral drinks which were prescribed for him, went to consult a physician in a village very near Perpignan. On examination, he found at the orifice of the pharynx, behind the velum palati, and at the left of the base of the uvula, a leech, about two inches long, and eight lines thick at its middle. It was extracted, after several attempts, with his thumb, forefinger, and a pair of dressing forceps. Dr. Bonafos, who relates the case, supposes that the blood proceeded from the body of the leech, squeezed by the action of the pharynx. The leech, having thus disgorged a part of the blood, was enabled to begin sucking again, which accounts for its not having dropped off.—*Journal des Connaissances Médico-Chirurgicales.*

### CASE OF STRANGULATED HERNIA FOLLOWED BY CHOLERA.

A robust grenadier, of temperate habits, was admitted into the military hospital at Antwerp, on the evening of the 5th of January, 1834. He wore a truss habitually, for an inguinal hernia on the right side, but he had taken it off the preceding night, on going to bed, and in the morning the hernia was down. It was harder and larger than usual, and he could not reduce it. He suffered from pain during the day, and in the evening was obliged to be carried to the hospital. The house-physician made new attempts to reduce the hernia, but did not continue them long, on account of the pain and tension of the parts. He took sixteen ounces of blood from the arm, applied sixteen leeches to the tumour and around the ring, and put the patient into a bath. After several unsuccessful attempts the tumour was reduced on the morning of the 6th. Meantime another set of phenomena had appeared. Vomiting had occurred during the bleeding, and had returned several times, and the patient had fallen into a remarkable state of debility attended with anxiety. These symptoms might be considered as the result either of the taxis or the bleeding; but the state in which I found the patient on the following morning, says Dr. Gouzée, was such as might make one doubt concerning their origin. His eyes had sunk into their orbits, and were surrounded by a bluish circle; his lips, hands, and feet were completely blue; the integuments pitted on pressure; there was vomiting of greenish matter, and extreme thirst; no stools, no urine, no pulse at the wrist; colic pains, but no cramps. The treatment consisted of mustard poultices to the legs and chest, an emollient cataplasm to the abdomen, and a table-spoonful every quarter of an hour of a mixture, consisting of seven ounces of peppermint-water, an ounce of syrup of marshmallow, and a drachm of sulphuric ether. On the 8th, he was well, with the exception of the thirst. He was discharged on the 21st.

Dr. Gouzée is of opinion, that in this case the sympathetic nerve formed the connecting link between the two diseases, and thinks it not improbable that the Asiatic cholera is essentially a disease of the sympathetic. He mentions a case which he saw in 1831, where a sabre wound in the abdomen was followed by blueness of the face and lips, alteration of the features, anxiety, loss of voice, and absence of pulse; and another in the Archives Génerales for November, 1833, where a man had been wounded in the chest by a foil, beneath the third rib. When visited by his physician, the patient seemed moribund; his skin was cold, his countenance blue and depressed, his pulse almost imperceptible, and he had a strong desire to make water, without being able to do so. He was better the next day, and out of danger the following one. The author of the case, M. Lesauvage, attributes these symptoms to a lesion of the cardiac and pulmonary plexuses; and Dr. Gouzée agrees with him.

[From the *Observateur Médical Belge*, a new and excellent medical Journal, published at Brussels. We have now before us all the numbers from its commencement in April, 1834, to November; the original articles are good, the extracts and translations judicious, and it is by far the best looking of the continental journals.—Ed. *Med. Quart.*]

### CASES OF HYSTERICAL AFFECTION OF THE KNEE.

Case i. A young girl, æt. nineteen, was admitted into St. George's Hospital in January, 1828, complaining of pain in the knee, which came on suddenly, three months previously, and prevented her from walking about. The pain was more severe some days than others, but did not prevent her from sleeping eight or nine hours every night. Cupping, leeches, and blisters had been employed without advantage. On examination, the form and colour of the knee were but slightly altered from their natural appearance, the skin of the knee and adjacent parts was preternaturally sensitive to the touch. The patient's general health was good, and menstruation regular. She was ordered to take aperient pills occasionally; a blister was applied near the knee, and a discharge maintained from the sore. The patient was dismissed the hospital cured, at the expiration of twelve days from her admission.

Case ii. A young woman was admitted into St. George's hospital, October 1st, 1829, with pain in the knee, extending down the leg. The complaint had existed ten weeks, being alternately better and worse. Leeches had been applied without any beneficial result. At the time of her admission, the complexion was of a jaundiced hue, the tongue furred, and the menses irregular; the pain sometimes ceased, but generally became worse after walking, the skin of the knee and lower part of the thigh was excessively tender to the touch. The following medicine was ordered: ℞. Decoct. Aloes Comp. Mist. Camph. *aa* ʒvj. M. bis die sumend: spirit lotion to the knee. The lotion aggravating the pain, it was withdrawn, and a blister applied in its stead.

On the 14th October, the general health was improved, but there was no amelioration of the local symptoms; the mixture was continued, straps of soap plaster and a bandage were applied to the knee.

On the 17th, being no better, the mixture was left off, and the following prescribed: R. Tinct. Castorei ʒss. Mist. Camphoræ ℥iss. M. pro haustu ter die sumendo. ℞. Ext. Coloc. Comp. ℈ss. Ext. Hyoscyami gr. iv. M. fiant pilulæ, iii., omni nocte sumendæ.

October 20th. She felt scarcely any pain, and the morbid sensitiveness of the skin was much diminished. Medicine continued.

October 22d. She had a recurrence of the severe symptoms. Medicine repeated, and a belladonna plaster applied to the knee.

November 1st. Since the last date the knee was sometimes painful, at other times free from uneasiness, the pain has increased within the last two days. The former medicine, and the belladonna plaster, to be discontinued, and Ammon. Carb. gr. iii. Mist. Camph. ℥iss, to be taken three times a day.

November 4th. No better since the last date. The mixture to be continued. ℞. Tinct. Opii. Spirit. Rect. *aa* ʒii Mist. Camphoræ ℥vj. M. fiat lotio constanter genu applicanda.

November 6th. The lotion appeared to relieve the pain and acute sensibility of the skin. The same remedies were continued for several days, the symptoms subsided, and the patient was dismissed the hospital on the 24th.—*Mr. Lee, on Nervous Disorders.*

### CASE OF ADHESION OF THE PLACENTA TO THE FUNDUS OF THE UTERUS.

I was called to visit a black girl, who, I was informed, was in labour, but, on arriving at the house, was told "she had been delivered of her child three days before, but that the after-birth was still retained." I found her with a countenance expressing much anxiety; severe pain in the region of the uterus; a hot, dry skin; and her pulse about 120 in a minute, and very small. On raising the bedclothes to introduce my hand for examination, there escaped the most disgusting stench I ever encountered. I then introduced my finger into the vagina, upon which she sprung to the head of the bed, exclaiming, "Oh, you will kill me!" I immediately withdrew my hand, and asked what was the matter? She then told me "that the nurse had felt her so much that she could not bear to be touched." I explained the necessity which existed for an examination, and that it was impossible for me to render her any assistance unless she submitted. She then told me she would bear it. I again introduced my finger, and carried it to the os uteri along the cord, which I found so firmly contracted that I could scarcely introduce the end of my finger, and its edges quite firm, and yielding none to the pressure made upon it. I then held the finger of the right hand at the os uteri, and drew gently with the left hand the umbilical cord, in order to ascertain where the adhesion existed by the course of the cord. This I found to be at the fundus, and that very firmly. The case to me was a novel one. I never had seen or read of a case having occurred, and the only light thrown upon the subject is a paragraph in Dewees' System of Midwifery, where he says that he has never seen a case of adhesion of the placenta to the fundus of the uterus, and at the same time a contraction of the os uteri, but that he would suppose the secale cornutum might be advantageously used. We know the powerful effects of this remedy on the fibres of the uterus; we know that it produces strong contractions; then the question arises, will the contraction of the longitudinal fibres overcome the force of the circular? If so, the os uteri must dilate. I believed it would, and consequently gave fifteen grains of the ergot as a dose. Its effects were powerful, and continued some time, but without producing any other effect. In half an hour I repeated the dose, with the same result as the first, except that the contractions of the uterus were stronger. I repeated the third, fourth, and fifth time, with an interval of half an hour between each dose. Immediately after giving the fifth dose, making in all seventy-five grains, I had the pleasure to find the os uteri open, the adhesion give way, and the placenta delivered. The girl did well, with the exception of a mild phlegmasia dolens, which yielded to simple remedies.—*Dr. Worrell, in American Journal of Med. Science.*

### FUNGUS HÆMATODES.

Dr. Smith has given, in the *Baltimore Journal*, a case of this disease successfully treated. The patient was suffering from two tumours, one on the most prominent part of the malar bone, and the other over the centre of the sterno-clavicular articulation. Dr. Smith first employed leeches, cooling lotions, cathartics, low diet, and repose, but without advantage. He then prescribed the Tr. Iodin. in doses of ten drops three times a day, and a pill composed of two grains of aloes, two grains of bluepill, and half a grain of ipecacuanha, to be taken every night; and also the empl. hydrarg. to be applied to the tumours. An amendment took place in two or three weeks, and ultimately the cure was perfect. The author states that he is treating another case with the same remedies, that they are keeping the disease in check, and that he does not despair of their bringing it under control.

### LIGATURE OF THE AORTA.

This tremendous operation was performed last January, at the Cape of Good Hope, by Dr. John Murray. The details are given in the *Medical Gazette* of October 4th, 1834. The patient was a Portuguese sailor, æt. thirty-three, suffering from aneurism of the external iliac artery, which had existed for three months. The aorta was tied three or four lines above its bifurcation, and about an inch below the spot where the inferior mesenteric artery is given off. It is almost unnecessary to mention the result of the operation,—the patient died in twenty-three hours.

### ON BELLADONNA IN PERTUSSIS.

Dr. Jackson tells us (*American Jour. of Med. Sciences*, No. 28,) that, having determined to try the effect of belladonna in hooping cough, he gave it, in the winter of 1831, after the manner recommended by Dr. Kahleiss, *i. e.* in combination with sulphur and ipecacuanha, and with alternating doses of prussic acid. The belladonna was spurious, and the mixture inert. In December, 1832, the disease invaded the author's own family, and the following is his account of the first case.

"Our second child, a girl in her ninth year, had high fever, and her cough was so violent that the blood streamed from her nose at almost every paroxysm. This too was the mere catarrhal cough that precedes the spasmodic and more distressing form of the disease. She was bled once, purged gently, took tartar emetic in nauseating doses, with an abundance of linseed-tea: thus the fever was soon reduced, and the characteristic cough began to be developed. We then commenced with extract of belladonna, giving it simply triturated with water, in what doses we do not recollect, but it was given freely till the pupils were greatly dilated, and her vision so confused that she could not read large print. To reach this point did not require more than two days, when the complaint being plainly mitigated, the medicine was for the present omitted. In about twenty-four hours the pupils began to contract, and vision to become more distinct; the belladonna was therefore resumed, and given in larger doses than before. How long she took it, or how large were the doses, we do not now recollect; nor is this point material to our present purpose, as we shall treat of it particularly towards the end; but we do very distinctly recollect that the relief was altogether unexpected and incredible, for in a few days (certainly not more than ten from the time she began the belladonna,) the child was totally cured. We do not presume to say that she coughed none, but we do say that her fever was gone, her appetite ravenous, that she was able to attend to all her lessons, and that her cough, if she had any, would have passed unnoticed in any family."

Dr. Jackson's four other children were treated with the same remedy, and recovered with the same facility.

On the hooping-cough breaking out in the families of two gentlemen named Snyder, living seven miles from our author, he recommended the belladonna; but the results were different in the two families, for " it certainly requires some courage to give this medicine in the most efficacious manner; and this fact, it is presumed, will deter many from giving it as they ought. George Snyder was frightened by the panic of his family physician, and hence his children are still coughing, whilst his brother Henry, who possessed some medical philosophy himself, persevered, and performed a cure many weeks ago."

The medicine should be given till dilatation of the pupil is produced, when it must be discontinued, or administered in smaller doses. Dr. Jackson thinks that one sixth of a grain of the extract may be given to a child three months old, every three hours from sunrise to bedtime; a grain to a child two years old, and a grain and a half to one four years old. Our own opinion is, that, if the extract be very good, these doses are too large, and too frequent.

### TREATMENT OF NASAL POLYPI.

Mr. Joseph Dallaway, divisional surgeon of the Coast Blockade, states that, in seventeen cases of common nasal polypi, he has succeeded in curing them all without forceps, or any other mode of treatment than a solution of the Sulphas Zinci in water, in the proportion of from ℈ij. to ʒj. of the former, in ℥j. of the latter. This lotion, he states, is to be introduced up the nostril by means of lint, which has been well moistened with it, and the lint spread over the surface of the tumour, as far up as can conveniently be effected, by means of a probe or director. This lint must be kept moistened by dropping in the solution of zinc four or five times in the day, and then by removing it night and morning, to be replaced with a fresh piece of such moistened lint. Mr. Dallaway states that his seventeen cases were all cured of the disease within a fortnight, by this simple remedy. He does not state, however, that any of the cases enumerated were of a malignant nature, but merely the common soft polypus, as I understand him.

Mr. Dallaway first adopted this practice in polypus, he says, so far back as 1797; and aptly remarks, that it *may* be equally successful in certain cases of polypus uteri. I must say that, upon the receipt of this paper, I tried the remedy in my own practice at the Westminster General Dispensary, and in three cases of the soft common polypus I succeeded, within ten days, in removing the disease; and I understand from my colleague, Mr. Thomas Chevalier, that he was equally successful with one or two others.—*Mr. A. Copland Hutchinson, in Med. Gazette.*

### THE ITCH INSECT.

The detection of this minute animal has created quite a sensation among the *savans* of Paris. M. Renucci, a student of the Hôtel Dieu, has been the first to put beyond a doubt the existence of the genuine Acarus *scabiei:* he has, in consequence, beside the credit due to him for his success, netted three hundred francs, which M. Lugol, a few years ago, offered for, or rather wagered against, the discovery of the insect. All the descriptions hitherto given of the *A. scabiei*, by Bonomo, De Geer, Baker, Alibert, and others, have been inaccurate and fanciful: they would have better served to represent the mite of cheese. The true *A. scabiei*, like the mole, has its fore legs strongly developed, while its hind quarters are comparatively feeble; it is thus enabled to burrow under the cuticle, and to make a road for itself as it proceeds. Plans and drawings of the animal on a greatly magnified scale have been laid before the Institute by M. Beaude; and M. Raspail, whose skill in exploring minute objects is so celebrated, is engaged with his microscope in procuring further details.

We learn from a letter addressed by M. Renucci to the Academy of Sciences, that, in the year 1825, while he was attending his brother's practice in Corsica, he often had an opportunity of observing the method pursued by women of that country in extracting the acarus, usually called by them the *pedicello*. M. R., aware that the existence of the insect was denied by many eminent naturalists and physicians on the continent, took pains to ascertain the signs of its presence: he devoted a good deal of study to the subject, and thus was enabled recently to point out the true *A. scabiei* to M. Alibert, at the Hôpital St. Louis.

The proper mode of proceeding for the purpose is to examine the vesicles of an itch patient newly infected, and not yet under cure. At the base of any one of them will be found certain little furrows, going off in different directions, some towards the summit of the vesicle, others running round it, and others again prolonged beneath the neighbouring skin. Observe the furrow which diverges most from the vesicle, and at its extremity will in general be detected a white point, visible with the naked eye. This white point, where the cuticle is slightly elevated, corresponds to the posterior part of the insect. In warm countries, says M. Renucci, I have further been able to discern the head, which is brownish. Whenever we can distinguish two such points, we may be almost sure of finding the insect. In order to extract it, we must pierce the cuticle with a needle, at about the distance of half a line from the white point, when, by gently dividing the epidermis upwards, the acarus is laid bare, and easily removed.

It is not rare to find it at the base of the vesicle; sometimes it is even found on the sides; but very rarely, or perhaps never, on the top. Most likely it was owing to this circumstance that many of those who sought the acarus scabiei with such industry *in* the vesicle, or its fluid, were induced, from their want of success, to deny its existence altogether. The figure given by De Geer is, according to M. Renucci, rather that of the itch-insect of the horse than of the *A. scabiei* of man.

But, after all, says a writer in the *Journal Hebdomadaire*, it does not follow that this insect, the presence of which we have ourselves witnessed, is the real cause of the itch. It still remains to be seen whether there may not be genuine itch without an insect; whether the acarus now detected be the same in all patients affected with scabies; and whether it be not found in other animals, or even in animal or animalized substances, placed in circumstances favourable to the propagation of insects, such as heat and humidity.—*Med. Gazette.*

### CASE OF UNUNITED FRACTURE, SUCCESSFULLY TREATED BY FRICTION.

Samuel Sapp, a stout, athletic man, aged about twenty-seven years, from New Jersey, applied to my father, during the last summer, concerning an ununited fracture of the humerus. He stated that, on the 1st of March, 1833, while engaged in his occupation, as one of the deck-hands on board the steam-boat Trenton, he was dragged overboard by becoming entangled in the rope, and, in attempting to save himself by seizing the railings, his left arm was fractured.

He immediately applied to a surgeon in a neighbouring town, who carefully adjusted the fragments, and placed the limb in splints; the injured parts being but slightly painful, the first dressings were allowed to remain undisturbed for about three weeks, when other splints were substituted, and continued on the limb, with occasional alterations, for three months. At the end of this period, finding no improvement, his physician advised him to seek further advice.

On removing the splints, the limb was found to be much reduced in size, its muscular power was obliterated, and its capillary circulation feeble. He was advised to lay aside the splints and bandages, to use the limb moderately, and to keep up a steady system of external frictions, and requested to return to the city in cool weather.

On his return in the autumn, no improvement was manifest; and, by the advice of my father, he placed himself under the care of my friend, Dr. William Ashmead, and myself.

On a careful examination of the parts, we found an unusual obliquity in the fractured portions, the surfaces exposed being not less than three inches in extent, the edges of these surfaces, the rounded extremities of the fragments, and the crevice separating the opposing surfaces of the fracture, could be distinctly traced by the fingers. This examination was rendered peculiarly satisfactory, in consequence of the emaciation and flaccidity of the limb.

Owing to the remarkable extent of the fracture, and the loss of muscular power in the arm, the fragments, which in a more vigorous state of the surrounding parts, might have been kept in apposition, were separated from each other to a greater or less extent, as they were influenced by the position of the limb. When the forearm was flexed upon the arm, in the usual attitude for fractured humerus, the surfaces of the fragments were separated throughout their whole extent, but more particularly at their upper portion, and it was only in one position that their apposition was effected.

The limb being placed in that position which we found upon trial effected a perfect coaption of the parts, the upper and lower portions of the broken bone were grasped by the hands, and a firm, gliding motion communicated, so that the surfaces could be felt rubbing upon each other. This process was continued for several minutes; and the limb was then secured in this position by light dressings in an angular box, a piece of thin board being firmly bound over the seat of fracture.

This process was repeated for several successive mornings, and was performed by Dr. Ashmead or myself: the few first trials excited but little sensation in the fractured surfaces, though the force used was as great as we could command. In a few days, however, the patient began to feel pain, which increased at every repetition of the process, until it became acute. The fractured ends were less moveable, heat and action were reestablished in the limb, and we were obliged to diminish the frequency and severity of the friction.

In about a month, bony union became evident at the lower extremity of the fracture, which proceeded rapidly, and so agglutinated the lower portion as to prevent the necessity of the box: shooting pains were frequently experienced in the limb, and any attempt to disturb it produced considerable suffering. Under these circumstances we declined interfering with the salutary operations of nature, which proceeded most happily. In about two months after the commencement of the practice, we had the satisfaction of observing that a firm bed of callus was thrown out over the whole surface of this extensive fracture.

The muscles soon acquired their accustomed volume and force, and the man has since been pursuing his laborious occupation.—*American Journal of Med. Sciences.*

### EFFECTS OF THE SOLUTION OF MURIATE OF ANTIMONY IN CARCINOMA. BY DR. A. NEUMANN.

G., a peasant, from the village of Grabowiez, was operated on for a carcinoma of the lower lip and right cheek, without any hope of being able to effect a cure of the wound by the first intention, on account of the great extent of the disease. The carcinoma had not only seized upon the lip from one corner of the mouth to the other, partly destroyed it, partly hardened it and bent it outwards, as well as changed the appearance of the soft parts as far as the bone of the lower jaw, and to the very point of the chin, but had also gone on from the right corner of the mouth over the thin surface of the cheek for about an inch, while the outermost parts appeared undisturbed. By the operation, the whole lip, beginning from the right corner of the mouth down to the bone of the lower jaw and the chin, and again up to the left corner of the mouth, was cut off; the right cheek was turned inside out, and, a second circular cut being made, the scirrhous tumor was separated from it. Upon an examination of the cut surface, it was found that it was entirely free from the hard carcinomatous parts, except in one place near the chin, and close to the maxillary bone, where a piece, about the size of a silver groschen, still remained.

Although the patient had as yet suffered very little, still he could not be prevailed upon to allow the knife to be applied again to the diseased part. Indeed, he frustrated even the attempt which was made without his knowledge to apply a hot iron before bandaging the wound, for which preparations had been made; he pushed aside three men who were holding him, and put himself on his defence against the author.

In this case, as the wound showed full three inches in its lower part next the chin, and from three to four inches in the upper part, and at the same time, as above mentioned, still contained carcinomatous parts, it was only possible to promote union by strips of adhesive plaster and bandages, and to effect a cure by granulation, the author intending to remove the carcinomatous parts afterwards by caustics. This was tried on the following day when the patient had become quieter, when the bandage had been removed, not by means of arsenic, but by applying a few drops of solution of muriate of antimony. This caused a blackish ichorous discharge on the carcinomatous spot, and for six days large and small pieces of the carcinoma, which reached to the maxillary bone, came off, after which healthy suppuration took place. By degrees the wound healed up on all sides, Ung. Basilicum alone being applied, and in about three weeks it was half its former size, without any great ptyalism having taken place, which was the chief thing to be feared. In eight weeks the wound had completely healed, whilst a lip, reaching up to the crowns of the teeth, had formed itself, by which means the patient could retain his saliva.

It was remarkable, that, in the whole lower part of the wound, and even in the cauterised carcinomatous place, where the whole of the soft parts had been removed as far as the periosteum, that as the scar made progress so black hairs sprung forth, which certainly in this case did not come from bulbs of hair left behind, as, in that case, within the periosteum.

I explain this appearance through the standing law of nature to work according to a fixed original model, and to regenerate a given organ on its appointed spot, as far as the strength of any particular class of animals will allow. This is the more probable, as in our case the new hair was of a handsome black colour, while the rest of the patient's hair, whether his beard or on his head, had become either grey or white, on account of his advanced age (fifty-eight.)

I cannot make my readers too attentive to the permanent extirpation of the cancer by the solution of the muriate of antimony, especially in these times, when it has unfortunately become so much the fashion to run after new remedies, and to overlook old ones, of which the effects are not yet sufficiently known and inquired into. I believe that, in most cases of carcinoma, arsenic, which is so dangerous on account of its being absorbed so easily, so painful, and so troublesome, might be replaced by muriate of antimony, even before the operation; and it is therefore to be desired that frequent trials should be made with this remedy in cancer, and the experiments, when collected, should be published.

I cannot refrain from pointing out the following rules for the application of the solution of the muriate of antimony. The remedy must be applied to the diseased part alone with the greatest accuracy, and this must be done once or twice a day. By this, if the surface was dry before, a dry, black, and as it were carbonized and hard crust, is formed; but, if it was a suppurating surface, a blackish ichorous crust, which is carbonized only in two or three places. If this does not come off of its own accord, it must be removed by means of careful incisions in the dead part, pushed as deeply as possible. The caustic is now to be applied again, and continued until the surrounding healthy parts begin to inflame, although untouched by it. This is a proof that the diseased part only is destroyed, and that the caustic has reached the healthy parts. Cicatrization must afterwards be promoted, and completed, by means of digestive ointment.

### CARBONATE OF AMMONIA IN THE URINE.

I was the first, I believe, several years ago to announce the discovery of carbonate of ammonia in urine recently voided, and that in considerable quantity, causing the fluid to effervesce briskly on the addition of an acid. The observation did not excite the attention, if it met the eye, of Dr. Prout and others who have since written on the composition of the urine in disease. As a second case of the kind, however, has very lately come under my notice, I think it well to return to the subject. The case, the particulars of which I formerly published, was that of a young man labouring under long-continued fever, attended with petechiæ. The urine contained carbonate of ammonia for four or five days, at a time he was extremely bad. As he improved this salt disappeared. We at first thought it might have been formed in consequence of the urine undergoing decomposition in the bladder; but it was proved that this was not the case, for when the bladder was completely emptied, the urine formed in it in two hours afterwards was found equally loaded with the same salt. There was no disease of the mucous membrane of the bladder whatsoever, and we therefore were justified in concluding that the carbonate of ammonia existed in the urine as secreted by the kidney. Although I afterwards examined the urine of numerous fever patients, I never met with the same salt. The case now under our observation at the Meath Hospital is very different indeed in every thing but the presence of this salt in the urine. A strong and athletic man, employed by the Ballast Board as a labourer, had occasion to work several days standing up to his knees in water. Being at the time constipated, he took a large dose of Glauber's salts which acted briskly on the bowels, but he did not cease to work in the cold water notwithstanding. The consequences of his imprudence soon became apparent, for the purgative effect of the medicine was scarcely over, when he was attacked with most violent pain in the belly, accompanied by great distention of the stomach and bowels, thirst, headach, and fever. In a few days he was admitted into the Meath Hospital, labouring under anasarca, ascites, and intestinal tympanitis. Bleeding, leeching, and the most active antiphlogistic treatment, greatly abated his sufferings, and diminished the intensity of the disease, but I fear all our efforts will prove unavailing to procure his final recovery. At the period that the pain and tenderness of the belly, together with the character and frequency of the pulse, demanded the first application of leeches, I was very much surprised to hear from Mr. Knott, a most diligent and intelligent pupil, that the urine contained carbonate of ammonia in considerable abundance. It was examined in Dr. Apjohn's laboratory by Mr. White, and was found to effervesce briskly on the addition of the mineral acids.

This appearance was owing to carbonate of ammonia in great excess. It was of rather a pale straw colour, contained no albumen, and acted on the vegetable colours as an alkali. It deposited a precipitate, consisting of the ammoniaco-magnesian phosphate and phosphate of lime. This remarkable urine was supposed by some, who witnessed the violence of its effervescence on the addition of an acid, to owe the formation of its ammoniacal salt to decomposition during its retention in the bladder. But that this was not the source of the carbonate of ammonia, was proved by many circumstances. It was perfectly limpid when voided, and had not the slightest smell of putrescence, such as exhales from urine even in the commencement of decomposition. Again, when our patient completely emptied the bladder of its contents, and in half an hour afterwards again passed a small quantity of water, this latter was found as copiously loaded with carbonate of ammonia as the former. It necessarily follows, therefore, that the urine, as secreted by the kidneys, contained the carbonate of ammonia which seemed to be a vehicle for excreting those elements which are usually combined so as to form urea, for in this man's urine not a trace of urea could be discovered.

The occasional presence of ammonia in the urine, in the form of the ammoniaco-magnesian phosphate, has been long known to chemists; carbonic acid is of much rarer occurrence indeed, for not more than one or two cases have, I believe, been observed, in which carbonate of lime has been found forming a urinary calculus in the human bladder, although so common in swine and other animals.

P. S. July 5. The post-mortem examination of this man exhibited the kidneys rather enlarged, and somewhat turgid with blood, the bladder perfectly healthy. The liver misshapen, round at the edges, smaller than natural, indurated, and composed throughout its whole mass of globular masses, very firm and pale, forming a variety of what is called schirrous liver.—*Dr. Graves, in the Dublin Journal.*

ACCOUNT OF A TRIAL OF ACUPUNCTURE WITH GALVANISM, MADE BY DR. W. STOKES, ONE OF THE PHYSICIANS TO THE MEATH HOSPITAL. BY JOHN HAMILTON, L.R.C.S.I.

With the expectation that the efficacy of acupuncture would be increased by passing galvanic shocks through the needles, such a combination was tried in France a few years since, and with apparently favorable results. But in this country, although the use of acupuncture is pretty generally known, and its efficacy admitted, I am not aware of any account of its having been tried with galvanism; the following sketch, therefore, of the result of some cases, in which such a trial was made by Dr. Stokes, in the Meath Hospital, will at least possess the merit of novelty.

The galvanic battery employed was a trough, with fifty-two inch zinc and copper plates, and the liquid, sulphuric acid and water, in various proportions, most commonly three drachms of the acid to eight ounces of the water.

The application was as follows. If a patient presented himself with paralysis of the deltoid muscle, from rheumatism or other cause, one needle was inserted tolerably deep into the upper fleshy part of the muscle, and a second at the lower part, so that, on applying the wires from the ends of the battery to the needles, the shock was passed quite through. Two other needles were occasionally passed at the sides of the muscle. Nine or ten shocks were given at a time, and repeated daily as long as necessary. As the strength of the shock depended on the number of plates used to complete the galvanic circle, that number differed according to the part and the susceptibility of the patient. In sciatica, for instance, where the parts about the seat of the disease are chiefly muscular, the shock from the whole fifty was sometimes passed along the limb. The employment of so great a number, however, was rare, from the violence of the shock being far too great, and anything near that number, where the galvanic fluid had to pass through a delicate or important organ, such as the brain or eye, was never used. The necessity of caution in this last respect is well shown in the following case. A man, formerly a soldier in India, with some obscure chronic disease of the brain, which rendered both eyes amaurotic, was ordered to be subjected to this agent; more from a wish to give him every chance where all the usual remedies had been tried in vain, than with much hope of success. The left eye being the worst, one needle was inserted a little above the left eyebrow, another at the lower part of the occiput at the same side. It had been determined to commence with only eight or nine pair of plates, but, from a feeling of caution, the circle of communication only included three pair. And yet, with this exceedingly small number, he was completely stunned for an instant, as if from the blow of a heavy stick, and felt a severe darting pain through the head, with a flash of light before the eyes. Now, where so severe a shock resulted from so few plates, and as we know the severity is proportioned to the number, it does not appear unreasonable to suppose, that, had the number commenced with been twenty-five, which would have given a shock upwards of eight times as strong, some severe lesion of the brain, or even death itself, might have been the result. In all subsequent cases of amaurosis, to prevent the likelihood of any injury to the brain, the place of insertion of the needles was altered, and consequently, the course of the galvanic fluid.

The extent to which the impression of galvanism on the system is increased by the aid of needles, and how much can be done by a small battery, is very remarkable. On merely applying the ends of the wires to the skin, no effect is produced, the galvanic fluid appears diffused over the surface and lost; but the instant they touch the needles inserted into a part, a most violent shock is given, making the stoutest cry out from pain, powerfully convulsing the muscles, generally throwing the patient into a profuse sweat, and often followed by faintness and sickness of the stomach. Some, from its severity, would not submit to a second operation. In a case of amaurosis one needle was inserted into the nasal end of the eyebrow, the others into the temple, by which means the shock passed in a great measure through the orbit, engaging the brain only to a slight degree. The patient was sensible of a disagreeable stunning feel in the forehead, with pain, the brows were reddened and contracted, a flow of tears came from the eye, with the sensation of a flash of light; and in one person in whom it was used by the late Mr. Hewson, going as high as twenty-five pair of plates, the pupils were observed to be very much contracted after its use, and a stinging pain felt in the head the rest of the day. In another case also, where the application was to the side of the face, for paralysis of the portio dura of the seventh nerve, rigors, with headach, and heat and prickling in the part, followed several times.

This great increase of power from the use of the needles evidently depends on their conducting the galvanic fluid beneath the cuticle, the well known property of which is to resist its admission. They accomplish what Humboldt effected by removing the cuticle by blisters, but in a much simpler, more expeditious, and convenient manner; giving scarcely any pain, if rightly introduced, and leaving no lesion behind them. The following circumstance attending their extraction is curious. In simple acupuncture all the needles inserted are generally withdrawn with some degree of difficulty; this difficulty was always found to be greatly increased after the application of galvanism in one of the needles, while the other was exactly the reverse, being invariably withdrawn with unusual facility, greater even than the natural consistence of the flesh would seem to permit. Some idea may be formed of the degree of resistance occasionally met with in the extraction of one of the needles, when I mention, that one gentleman found it so great, that after exerting much force in vain, the needle still sticking tightly in the lumbar muscles, he expressed his conviction that it must by some means have got bent, and thus hooked in.

The needle difficult of extraction was always that to which the positive wire had been applied, the other, that touched by the wire from the copper or negative end of the battery.

This phenomenon appears to depend on the power which the positive end of the battery is known to possess of giving irritability, and that of the negative in taking it away. "If silver be applied to nerves and zinc to muscles, the irritability of the latter increases in proportion to the time they have remained in the chain. By this method the thighs of frogs have been revivified in some degree, and afterwards become sensible to stimuli that before had ceased to act on them. By distributing the metals in an inverse manner, applying zinc to nerves and silver to muscles, an effect absolutely the contrary is observed, and the muscles that possessed the most lively irritability, when placed in the chain seem to be rendered entirely paralytic." Applying these principles to the facts before us, we can readily conceive how the muscular fibres round the positive needle, endued with a tenfold degree of irritability, tightly grasp the needle, and resist its removal, while the exhausted and paralysed fibres round the negative needle yield it up to the slightest force. Wishing to see how far these effects extended, I inserted the needles scarcely a quarter of an inch apart, but still found the positive needle hard, the negative easy to extract. On changing the application of the wires, so that each needle was touched by both, both needles were equally resisting.

Between the needles there was generally a red blush, and the shock extended no farther; in one instance, however, where one needle was inserted on the outside, the second on the inside of the right ankle, a strong convulsive twitch was excited, extending up the leg and thigh, and slightly convulsing the right arm and temporal muscle, accompanied by pain in the same direction.

As a remedial agent, I regret to say, the cases in which this combination of acupuncture with galvanism has been tried leave little to be said in its favour. Even were its efficacy greater, the application is so severe as to preclude its use, except in cases of a hopeless character, and where milder means had been resorted to in vain. It was tried in the manner described above in numerous cases of chronic rheumatism, several of sciatica and lumbago, a few of amaurosis, and some of paralysis, two of which were from lead. A case of lumbago and paralysis from lead were certainly cured, after many other remedies had been tried in vain, and a case of rheumatism greatly relieved; the most striking instance, however, was a case of paralysis of the portio dura of the seventh nerve, which I think well worthy of giving at length, as presenting a very accurate picture of this very interesting and rather rare form of disease, as well as a full exemplification of Sir Charles Bell's views on this subject. It was taken by my friend, Mr. Ellison, of Liverpool, who applied the galvanic shocks himself.—***Dublin Journal.***

### CAPIVI IN CATARRH OF THE BLADDER, AND LEUCORRHŒA.

The following cases, in which this remedy was successfully employed, are given by Dr. La Roche in the 27th Number of the *American Journal.*

Case i. A French gentleman, aged sixty years, who, after two attacks of acute inflammation of the bladder, was suffering from a muco-purulent discharge, was cured in a few months by the capivi, during which period it was several times necessary to discontinue the medicine.

Case ii. A gentleman, aged about sixty-five, whom Dr. La Roche saw only once, was then labouring under chronic inflammation of the bladder, and the author therefore prescribed leeches, tepid baths, &c., recommending the capivi to be taken when the inflammation was subdued. This was accordingly done, and the patient was very much relieved, indeed, almost cured. The ultimate termination of the case was not so fortunate.

"This gentleman suffered some time after a severe relapse, and had recourse to the same means, and with an equally happy result. After enjoying a tolerable share of health during two or three years, he again became a sufferer from disease of the bladder; the obstruction in the urethra increased gradually, and at length became almost unconquerable, except by mechanical means; the pain became more and more severe; the discharge of mucus from the bladder increased, was attended with pain, and effected with considerable difficulty. In this condition he removed to Philadelphia, and in the spring of 1829 once more placed himself under my care.

The usual remedies for complaints of that kind were resorted to. under the direction of two of the ablest surgeons of the city and myself, but were not productive of the least amelioration. The patient wasted away; and, after suffering unexampled agony during several months, sank into the grave. On examination after death, an ill-conditioned ulcer of the bladder, (in the centre of which was found a small calculus,) was discovered. The prostate gland was slightly enlarged."

Case iii. Mr. R., about fifty years of age, was seized with inflammation of the bladder, after an attack of gonorrhœa. When the acute symptoms had subsided, tonics, chalybeates, and terebinthinous remedies, were tried without advantage. The balsam of capivi was then prescribed, but at first disagreed with the stomach; laudanum was added to it, and various vehicles tried, among which weak claret and water was found the best. When this difficulty was got over, the capivi was of admirable efficacy, and cured the patient in a fortnight.

Case iv. *Irritability of the Bladder.* Mr. G., æt. forty-five, of an amorous temperament, was labouring under incontinence of urine. He went through a variety of treatment without benefit, but was cured in a few weeks by the balsam. Dr. La Roche has since cured a similar case with greater rapidity; and Dr. Hays has had the like success.

Case v. A coloured woman, æt. thirty, was suffering from inflammatory leucorrhœa, which was treated with antiphlogistic remedies. Her general health improved, and the fluid discharged, though still abundant, was no longer so dark, or so viscid. Twenty-five drops of the capivi were now exhibited three times a day in a wineglassful of milk: the patient was much better in a fortnight, and cured soon after.

Case vi. Madame B., æt. forty, complained of dyspepsia, inflammation of the bladder, and leucorrhœa. The inflammation was cured by antiphlogistic treatment, and the dyspepsia by prussic acid; nothing remained to combat but the leucorrhœa. This was accomplished by the balsam, but with some difficulty, for the remedy at first caused pain, nausea, and occasional vomiting. The capivi was taken for a month with great advantage, and the cure was completed by astringent lotions.

Case vii. Mrs. B., a native of New Orleans, æt. twenty. In this instance the leucorrhœa had been aggravated by astringents. The patient refused to lose blood, and therefore the treatment for the first ten days consisted only of a more slow and less efficient means, such as a low vegetable diet, acidulated and emollient drinks, saline purgatives, rest in a horizontal position, emollient vaginal injections, and tepid baths. The capivi was then administered with great relief, though the patient was several times obliged to discontinue its use, from the gastric irritation which it occasioned.

[We agree with Dr. La Roche that the capivi is an admirable remedy in several diseases; and the chief reason that it is not more frequently administered, is the difficulty of accommodating it to sensitive palates and irritable stomachs.]

### ON THE DIAGNOSIS OF FRACTURES OF THE NECK OF THE FEMUR.

There is a well-written paper on this subject, by Mr. Robert William Smith, in the last Number of the *Dublin Journal.* When speaking of shortening, the author says,

" In the museum of the Richmond Hospital there are fifteen examples of fracture of the cervix femoris; thirteen of these are taken from patients who died in the hospital; the degree of shortening in each case was carefully noted, and every precaution taken to insure accuracy of measurement. The following table shews the age and sex of the patients, the situation of the fracture, and degree of shortening.

| *No* | *Age.* | *Sex.* | *Situation.* | *Shortening.* |
|---|---|---|---|---|
| 1 | 36 | Male | Internal. | $\frac{3}{4}$ Inch. |
| 2 | 48 | Do. | Do. | $\frac{1}{2}$ |
| 3 | 74 | Do. | External. | $1\frac{1}{2}$ |
| 4 | 80 | Female. | Internal. | $\frac{1}{2}$ |
| 5 | 80 | Male. | External. | 2 |
| 6 | 70 | Female. | Do. | $1\frac{1}{2}$ |
| 7 | 75 | Do. | Internal. | 1 |
| 8 | 80 | Do. | Do. | $\frac{1}{2}$ |
| 9 | 60 | Do. | Do. | $1\frac{1}{2}$ |
| 10 | 82 | Do. | External. | $\frac{3}{4}$ |
| 11 | 78 | Do. | Internal. | $\frac{1}{4}$ |
| 12 | 80 | Do. | Do. | $\frac{3}{4}$ |
| 13 | 90 | Do. | Do. | $\frac{1}{2}$ |

" From this table it appears that, with the exception of No. 9, the shortening did not exceed one inch in any case of intracapsular fracture, and reached that extent in only one instance; nor, with the exception of No. 10, was it ever less than one inch and a half in the extra-capsular fracture. With respect to No. 9, the fracture, at the time the measurement was made, had existed for years, and the neck of the bone was absorbed, so that the degree of shortening was of course considerable. With respect to No. 10, I shall relate the particulars of the dissection.

" The neck of the bone was broken at its base, external to the capsule, and was forced a short distance, about three quarters of an inch, into the cancelli of the shaft, between the two trochanters; it was firmly impacted, and the line of fracture remarkably obscure; the trochanter minor was broken transversely, the line of fracture passing at right angles with the shaft of the bone, but without detaching any part of the process; the descending ramus of the pubis was broken obliquely, the fracture passing downwards and inwards from the thyroid foramen. The patient, a female, ætat. eighty-two, was thrown down by a cart loaded with hay; the horse and cart were stated to have both passed over her. She died upon the fourth day after the injury.

"In this case the slight degree of shortening may, I think, be fairly referred to the manner in which the fragments were disposed of with regard to each other, and the firmness with which the upper was impacted into the lower, by which muscular force was completely counteracted. It seems probable that the difference of opinion which exists upon this point, is in some measure owing to a proper distinction not having been made between the shortening which comes on during the first two or three days after the accident, the result of muscular action, and removable by extension, and that which we notice at a later period, the consequence of absorption, and permanent. Indeed, the period at which shortening occurs is subject to great variety: it may manifest itself instantaneously, immediately after the receipt of the injury, and that to a considerable degree; in such cases I have generally found a comminuted fracture external to the capsule. Sometimes the shortening does not become evident, until perhaps five or six days after the accident: in such cases the muscles have been paralysed by severe contusion; according, however, as they recover their tone, the limb is slowly shortened, and this independent of any process of absorption. Again, there are cases in which the retraction, slight at first, becomes, at the end of a month or six weeks, considerable. No. 11 in the table was an instance of it; the shortening, which at first was only a quarter of an inch, at the expiration of six weeks amounted to one inch and a half. It was found after death (which took place about two months after the accident,) that the neck of the bone was absorbed. Lastly, there are instances in which the limb retains its natural length for many weeks, and then becomes shortened, not gradually, but suddenly: these are the cases in which diagnosis has been found so very difficult. The cause which has produced the fracture is, in general, comparatively slight, and the patient has made no attempt to walk after the receipt of the injury. The eversion of the foot is by no means so well marked as when the shortening occurs early; nor is there much, if any, change in the position of the trochanter. The patient, no doubt, is unable to raise the limb *en masse;* but from this we can draw no certain conclusion, as it may be owing to a paralysed state of the muscles. If we can ascertain crepitus, the diagnosis is no longer difficult; if we cannot, we must only watch the progress of the case attentively, and if, after a period of two, three, or four weeks, the powerless state of the limb continues, we have reason to suspect some more serious injury than contusion. The eversion of the foot is now more clearly marked, and now also it frequently happens that the limb becomes shortened suddenly. The knowledge of this fact is not unimportant, as it usually indicates a fracture within the capsule. In the first case in which I

observed it, it took place at the end of three weeks: in the second, after the lapse of six, when the patient, having left his bed, and attempted to walk, the limb (which up to this period retained its natural length,) became suddenly shortened. The circumstance has been observed by Sabatier, occurring twenty-four hours after the accident. In these instances we must suppose that, at the time of the accident, the close coverings of the neck of the bone having escaped without injury, prevented the retraction of the limb, but that subsequently they were torn, either in consequence of some imprudent exertion on the part of the patient, or too eager a desire on that of the surgeon to ascertain crepitus by powerful extension and rotation of the limb: the retraction then takes place as the immediate consequence of their laceration."

He terminates by the following deductions:

"1. The less the degree of shortening, the greater is the probability that the fracture is within the capsular ligament.

"2. The degree of shortening, when the fracture is within the capsular ligament, varies from a quarter of an inch to one inch; when external to the capsule, from one inch and a half to two inches and a half.

"3. The limb may remain without any change in length for many weeks after the receipt of the injury; then retraction may suddenly occur.

"4. This sudden retraction, at a period more or less remote from the receipt of the injury, indicates a fracture within the capsule.

"5. The presence of morbus coxæ senilis may not only lead us to suppose that a fracture exists, when the bone is entire, but also when there is no doubt as to the existence of fracture, may render diagnosis difficult as to the seat of the injury with respect to the capsule.

"6. Inversion of the foot may accompany fracture within the capsular ligament.

"7. The accident most liable to be confounded with dislocation upon the dorsum ilii, is fracture through the trochanters, together with inversion of the foot.

"8. The degree of shortening, when the fracture is within the capsule, chiefly depends upon the extent to which the fibrous reduplications have been torn.

"9. When the hip-joint, long affected by that disease peculiar to old people, receives a severe contusion, we distinguish the accident from fracture of the neck of the bone, by the impossibility of restoring the limb to its natural length by extension, and also by an inquiry into previous history."

### INSTANCE OF DESTRUCTION OF THE UTERUS, PERINEUM, AND RECTUM, AFTER DELIVERY, WITH RECOVERY.

By Dr. John Sweet, of Ridgway, New York.

A woman, aged thirty years, of robust constitution, was delivered of her first child on the 28th day of June, 1830, full-grown, after thirty-six hours severe labour. During this preternatural labour blood was abstracted, and three or four doses of opium administered; forceps of a bad construction were unsuccessfully applied by a physician little acquainted with the use of instruments; the parturient pains immediately ceased, palsy and weakness of the lower extremities ensued, with pain in the back and hips. The patient was left in this situation by her attending accoucheurs, several hours, whilst they reposed on their beds! The child was born with little manual assistance, and without an effort of the parent.

On the 2d day of July I was called, and visited this unfortunate female, and found her labouring under very acute pain; pulse one hundred to the minute, vomiting a green and dark-coloured bile. Her friends had despaired of her life.

In this case I administered the sulphate of magnesia in a cathartic dose, which immediately prevented the vomiting, and in a few minutes she requested food, which being allowed her, was found agreeable to the stomach; and as often as the vomiting took place, the same medicine was administered, with the same effect as above stated.

On inspection I found the labia pudendi and posterior region in a gangrenous state; for this I prescribed yeast and charcoal, internally and externally, and also the cort. peruv. flav. acidulated with the sulphuric acid, together with wine to support the system.

The 12th day of the same month I found her labouring under tympanitis; I therefore discontinued the former medicines, and administered the ol. ricini. freely, directed ablutions of cold water, and a bandage to be applied to the abdomen; in a few hours the bowels were evacuated, a gentle diaphoresis appeared, and she was again relieved from pain.

On the 13th it was found that the fundus uteri had passed into the vagina, and out at the os externum: by a little assistance of the nurse, the whole uterine system separated, leaving its destined place of abode.

15th. The rectum parted a few inches above the pubes, and was discovered sliding down, and passing out between the labia pudendorum: the nurse, in my presence, took hold of and gently extracted it, the lower end easily separating from the sphincter ani, and without pain to the woman. At this time I proceeded to a further examination, and found the perineum destroyed, so much so as to leave but one orifice to the abdomen, (and that) extending from the os coccygis to the ossa pubis. The sphincter urinæ also had lost its power of contraction, and she labours under an incontinence of urine and fæces. She suffered several weeks with cruritis or phlegmasia dolens, partially submitting to the antiphlogistic plan of treatment.

In the month of November following, by an inspection, I found the pelvis to be healed, and could of a certainty discover the purulent discharge flowing from the abdomen. A portion of the intestine had visibly descended into the pelvis three or four inches, folded down, and hung pendulous over the sacrum in the vaginal cavity, and which was sensibly affected with cold air; for this a pessary of fine sponge was applied, and at length found useful in retaining the intestine in the abdomen, and in effectually excluding the cold air. So great was the destruction of parts, and so extensive the orifice, that the eye could trace the whole internal cavity of the pelvis so distinctly in this case that I had ocular demonstration, both externally and internally, that the rectum as well as the whole uterine system had separated, and were removed. Another fact of which I was informed, and had no reason to doubt, and which should by no means be omitted, is, that the fæces had ever, from the time of the above occurrence, passed off between the labia.

Notwithstanding all the sufferings of this patient in the entire loss of (these) important viscera, the great length of time she was sick, confined to her bed, and the continued inflammation of the abdominal viscera keeping up a purulent discharge, yet her strength, by the assistance of a remarkable appetite, held out, and health was fast returning.

About the 1st of January, 1831, the mammæ began to perform the office of secretion, so that milk flowed in profusion, and continued so about two months, and then ceased. This phenomenon happened six months after the extraction of the fœtus, apparently producing no material change in the other functions of the system, either by its continuance or cessation. Health now returned with bloom and vigour.

*February* 16*th*, 1832. I am informed by the father of this woman that she can now in some small degree command the urinary organs, and feels a sure confidence that in one year more she shall possess the full command: this is in my opinion possible, but over the fæces she can never have control. The inconveniences which she must ever for life sustain from incontinence of urine and fæces are very great; yet her health in every other respect is unexceptionably good to this day, viz. March 24th, 1833.—*American Journal of Med. Sciences.*

### CASE OF VARICOSE VEINS.

On the 14th August, 1833, we examined the body of a man who had died of cholera in the Saint Marylebone Infirmary. There was a large varicose ulcer situated over both the right and left tibia, and there were several branches of the saphena veins tortuous, and apparently greatly dilated, which extended above the knee.

We cut down to the trunk of the left saphena, where it was about to enter the femoral vein, and introduced the pipe of a syphon and threw water into the vessel, which immediately distended the trunk and all the varicose branches along the tibia. On removing the vein, its coats were found to be hypertrophied and thickened like those of an artery, and the inner surface of the vessel was rugous in the longitudinal direction. The valves were thin, pellucid, and perfectly healthy in appearance, though insufficient to close the vessel. Two enlarged and indurated glands pressed on the trunk of the saphena where entering the femoral vein. Around the cicatrix there was a large cluster of enlarged veins, and passing from these, through the fascia, was a large branch, which formed a communication with the deep-seated veins of the leg.—*Dr. Lee; in Cyclop. Pract. Med.*, Part xxiii.

### DIFFICULTY OF DIAGNOSIS.

I shall now point out to you several cases in which we may be led, by the very instruments we use for the detection of diseases, into an error in the diagnosis. I was consulted last winter by a very interesting patient, the brother of a medical friend at Fowey, in Cornwall: he had been taken with difficulty in swallowing; this difficulty gradually became greater, until the dysphagia passed into absolute inability to perform, or at least to complete, the act of deglutition; yet a certain portion of fluid could be apparently swallowed, as a small cupful; but, on adding to its quantity, it regurgitated through the throat and nostrils, and by an act precisely similar to that of vomiting. I took my patient to one of our first surgeons: he passed an œsophagus-tube, and the fluid recently swallowed flowed through it freely. It was concluded that the tube had entered the stomach, and, of course, that there was no obstruction to its course along the œsophagus. Nevertheless, the symptoms remained, and I was persuaded that, in fact, the act of deglutition, and the tube, had never passed beyond the œsophagus. The event confirmed my opinion. The patient emaciated, and died in eight weeks: on examination, the cardia was found completely obstructed by a morbid growth. If you will look at the drawing, you will understand what occurred in this case; the first acts of deglutition distended the lower part of the œsophagus; the subsequent ones filled this tube, until at length the fluid remaining in contact with the fauces irritated and excited the muscles which close the larynx and accomplish expiration, and produced the act of vomiting; the œsophagus-tube, and this œsophageal vomiting, merely emptied the lower and distended portion of this canal, without extending into the stomach.

A mistake is still more liable to occur in the use of the rectum-bougie. This instrument is apt to rest upon the promontory of the sacrum, in the manner represented in the drawing, giving great pain to the patient, and exciting in the mind of the surgeon the idea of stricture, or other obstruction. In other cases, the bougie catches a fold of the mucous lining of the intestine. A medical friend of mine, residing at Ollerton, in Nottinghamshire, fell into a state of indisposition, and remained nine weeks under my roof. I never suspected any disease of the intestine. He then went to Bath. In that city he consulted a young surgeon, who passed a rectum-bougie, and fancied he discovered stricture. The use of the bougie was continued daily for several weeks, giving great pain, and causing much irritation. I then met the patient in London; I saw no sufficient evidence of stricture, and I stated my opinion upon the case. I took the patient to Sir Charles Clarke, who, after an examination, confirmed that opinion. The bougie was relinquished, and from that time the factitious disease of the rectum, for such it was, was forgotten.—*Dr. Marshall Hall's Introductory Lecture.*

### CASE OF HEPATIC ABSCESS.

A case of this disease, in which paracentesis was performed, is narrated by Dr. Horner, in the 27th Number of the *American Journal.*

The patient was a tax-collector, aged fifty-five, suffering from hepatitis. There was an indurated swelling in the right hypochondrium, with evident fluctuation, but, as it was not clear whether an adhesion had formed between the liver and the anterior wall of the abdomen, an operation would obviously run the risk of producing a fatal inflammation, by allowing the pus to fall into the cavity of the peritoneum. Still, as the patient was sinking under the malady, it was necessary to encounter the possibility of this calamity; with what success the following quotation will show.

"The case being in this unpromising condition in every view of it which could be taken, a choice of evils only was left, and with the consent of the family, and the advice and assistance of Dr. Harris, the operation was undertaken, October 1st. An incision was first of all made horizontally on a line with the anterior end of the eighth rib on the right side, a little in front of its cartilage, and through the side of the abdomen, which brought the liver into view; the latter was seen to rise and fall with the diaphragm in respiration; moreover, a knife-handle was introduced between the surface of the liver and of the contiguous part of the abdomen; these two facts made clear the thing apprehended, to wit, want of adhesion. In this dilemma I determined to stitch the liver to the side, which was accomplished with a large crooked needle, armed with a ligature of kid-skin, and of bulk sufficient to fill up the hole made by the needle. One stitch was made in this way parallel with the upper margin of the incision, at the distance of four lines from it, and another in the same manner, below. The liver being thus fixed closely to the side, a trochar and canula were plunged into the abscess, and five gills of purulent matter were immediately discharged, to the great relief of the patient; the matter continued to flow during the night, so that three or four more gills were discharged. The operation being ended, a bandage was put around the abdomen, so as to keep its viscera as still as possible. The canula was left in for fifty-four hours, and then a piece of a flexible catheter was substituted; the abscess discharging all this time small quantities of pus and serum mixed.

"On the second day the bowels became tympanitic, and there was hiccup, with colicky pains. On the third day there was a manifest declension of strength, and it became evident that the previous exhaustion of the patient must render the operation nugatory. The symptoms of debility increased, and the patient died on the 5th inst. No sign of peritonitis followed this operation.

"In twenty-two hours after death an examination was made. A recent adhesion between the liver and side had occurred immediately around the puncture of the trochar, and which, along with the stitches, had prevented any pus from getting into the cavity of the peritoneum. The latter membrane was entirely sound, and had no appearance of being irritated by the operation. The cavity of the abscess was collapsed very much, and contained shreds of coagulating lymph mixed with pus, amounting in all to about one gill; its parietes were lined by a membrane. The right lobe of the liver being the seat of it, the anterior half was gone, but it appeared to be rather by pressure and absorption than by dissolution, as no remains of the liver were seen in the discharges. There was no preternatural adhesion of the liver to the parietes of the abdomen, excepting what was made by the operation. The left half of the stomach was destitute of mucous coat, it having been dissolved completely, so as to exhibit the cellular coat naked. Six inches of the beginning of the colon were studded with ulcers, having red, injected, and elevated edges. The small intestines were sound.

"Though life was not saved by this operation, evidently owing to the exhaustion of the patient at the time of its performance, I yet consider it as illustrating the fact, that hepatic abscess may be managed by opening it, even when adhesion to the side has not occurred; provided the liver be secured in the way described, or by an equivalent process; and, after a deliberate review of the case, I only regret that I did not resort to this treatment when the abscess first fluctuated."

### NOTES OF A CASE OF FISTULOUS OPENING OF THE STOMACH, SUCCESSFULLY TREATED BY DR. J. H. COOK.

Some time in the month of February, 1832, Dr. Bardwell and myself were called to visit the widow D., aged thirty-nine years. We found her, as near as may be, in the following condition: A fistulous opening, immediately by the side of the umbilicus, into which a buck-shot might have been readily passed; on removing the bandage, a gill of bile was suddenly discharged; after which, a small quantity of a different (the gastric?) fluid, came slowly away. These discharges were attended with great pain, on account of their acrid quality. The whole surface of the abdomen was excoriated, inflamed and intolerably painful. We introduced a flexible catheter its whole length, thirteen inches, before meeting with any resistance, when the extremity suddenly met with an obstacle. By pushing it against the resisting body, or even by slightly agitating the instrument, strong efforts to vomit were produced.

Withdrawing the catheter, we desired her to drink a glass of water; she did so, and in twenty seconds, the whole was discharged through the fistula, as we ascertained, by measuring it. The direction of the fistula was upward, and slightly inclining backwards, with about the same inclination to the right side. We came to the conclusion, that the opening within, was at, or about, the pyloric orifice of the stomach; and that the catheter entered the stomach, and pressed against its cardial portion. The patient even felt it there, and applied her hand externally over that part.

*Treatment.* Taking a large beef's bladder, we cut it open longitudinally, spread it well with adhesive plaster, and after washing the inflamed surface, and dressing it with basilicon spread on fine linen, we applied the bladder over the abdomen, and made an opening over the fistula, through which the matter might escape. We then applied a bandage and compress, and directed that it should be reapplied immediately after each discharge. We advised mucilaginous drinks, and a diet of rye-mush and molasses, and nourishing enemata. The patient was much emaciated for want of proper nourishment, as every thing passed off undigested through the fistula. No evacuation had taken place in the natural way for ten days previous to our visit. The external irritation of the abdomen soon healed, and the bladder was then applied to the skin as a protection, and continued there with the happiest effect. The bandage was gradually tightened, and a compress of a cylindrical form was laid over the course of the fistulous canal. By these means our patient regularly, but slowly, recovered. In a few days the alvine evacuations were restored to their natural outlet, and the discharges from the fistula began to decrease. In thirty days the opening was closed, and the fistula apparently obliterated. Several months have elapsed since that event, and she continues in excellent health.

All we could collect from her, as to the history of her case, was this: Six months before, in one of the south-east counties of this state, she was attacked with constipation and violent pain at the pit of the stomach, which resisted every remedy, till the 19th day, when the fistula showed itself.—*Western Journal of Med. and Phys. Sciences, January,* 1834; *in American Journal.*

### USE OF OPIUM IN MANIA.

When opium was prescribed by our judicious friend, Dr. Adair Crawford, while he was in charge of the Richmond Lunatic Asylum, Dublin, during the febrile stage of mania, either in small or large doses, with a view of quieting the excessive cerebral excitement and procuring rest, it always failed, and sometimes aggravated the delirium. It became evident, therefore, if benefit was to be derived from opium, that this could be expected only in the second stage, when there is no increased vascular action, and the maniacal delirium depends chiefly on cerebral irritation of a nervous character. No decided advantage even in this stage was obtained from opium, when given at night only, how large soever the dose. Then the plan of repeated doses throughout the day was tried, commencing with a grain every four hours, and gradually increasing or lessening the dose, according to the effect produced. Dr. Crawford soon observed that very large doses of opium could be taken without making the slightest impression on the delirium, and without any apparent effect on the system. Eight, ten, or twelve grains, were taken in the twenty-four hours, without affecting the state of the appetite, condition of the tongue, regularity of the bowels, or disturbing the circulation. It seemed as if the cerebral excitement protected the constitution from the ordinary influences of the remedy. By persevering in the use of cautiously graduated doses of opium in every case, the delirium was sooner or later overcome. The patient first appeared drowsy, and then became calm and rational. In some cases he suffered for a day or two from nausea, thirst, constipation, and vertigo, and the other usual effects of opium, which however soon disappeared. It is remarkable that the remission of delirium thus obtained was not merely temporary; in several cases the relief was permanent, and the patient left the hospital cured. In some the delirium returned after a remission of several days, but was again subdued by opium, and the paroxysm of mania was ultimately cut short. The quantity of opium borne was proportionate to the violence of the delirium; the largest dose to which the remedy was carried, was sixteen grains in twenty-four hours. It is remarkable, that while there was a great tolerancy of opium in the second stage of mania, there was an equally great tolerancy of tartar emetic in the first or febrile stage; and there is no more simple and effectual means of subduing the febrile action than by full doses of the latter remedy.—*Dr. Cheyne, in Cyclopædia of Practical Medicine,* Part xxiv.

---

